# Fast-Swelling Tamarind Xyloglucan/PVA Hydrogels with Interconnected Macroporous Structures for Biomedical Applications

**DOI:** 10.3390/polym16243457

**Published:** 2024-12-10

**Authors:** Umpornpan Ninjumrat, Piyachat Chuysinuan, Thitirat Inprasit, Sarute Ummartyotin, Kittipong Chainok, Penwisa Pisitsak

**Affiliations:** 1 Department of Materials and Textile Technology, Faculty of Science and Technology, Thammasat University, Pathum Thani 12121, Thailandthi_inp@tu.ac.th (T.I.); sarute@tu.ac.th (S.U.); kc@tu.ac.th (K.C.); 2 Laboratory of Organic Synthesis, Chulabhorn Research Institute, Bangkok 10210, Thailand; piyachat@cri.or.th; 3 Center of Excellence on Petrochemical and Materials Technology, Chulalongkorn University, Bangkok 10330, Thailand

**Keywords:** biocompatible polymer, capillary action, fast swelling, freeze-drying, hemicellulose, hydrogel, poly(vinyl alcohol), polysaccharide, tamarind xyloglucan

## Abstract

This work demonstrates the preparation of fast-swelling hydrogels based on poly(vinyl alcohol) (PVA) and tamarind xyloglucan (XG), utilizing freeze-drying to achieve an interconnected macroporous structure. Although XG is non-toxic and abundant, it has poor mechanical properties. Therefore, XG was mixed with PVA and crosslinked with citric acid (CA). Without XG, the crosslinked PVA sample contained partially aligned channels several hundred microns wide. The addition of XG (25% *w*/*w*) reduced the structural order of the hydrogels. However, the addition of XG improved the swelling ratio from 308 ± 19% in crosslinked PVA to 533.33% in crosslinked PVA/XG. XG also increased the porosity, as the porosity of the crosslinked PVA, XG, and PVA/XG samples was 56.09 ± 2.79%, 68.99 ± 2.06%, and 66.49 ± 1.62%, respectively. Resistance to compression was decreased by the incorporation of XG but was increased by CA crosslinking. The determination of the gel fraction revealed that CA crosslinking was more effective for the PVA component than the XG component. The swelling of all hydrogels was very rapid, reaching equilibrium within 10 s, due to the interconnected macroporous structure that allowed for capillary action. In conclusion, the prepared hydrogels are non-cytotoxic and well suited for biomedical applications such as drug delivery, wound dressings, and hygienic products.

## 1. Introduction

Xyloglucans (XGs) are mucoadhesive polysaccharides classified as hemicelluloses and are primarily found in the cell walls of dicotyledons and non-graminaceous monocotyledons, as well as in the seeds of certain tropical trees. Tamarind seeds, which are commercially available and abundant, serve as a rich source of this polysaccharide [1]. The tamarind XG backbone comprises β-1,4-D-glucose units, with approximately 70% of the glucose residues substituted with α-D-xylose through α-1,6 linkages. In some instances, β-1,2-D-galactose may be attached to the xylose residues, with fucose linked to the outer galactose residues. XG molecules typically form ribbon-like, twofold helices with an average molecular weight exceeding 50,000 [2,3,4,5,6]. It is water-soluble, non-toxic, and biocompatible, and has been FDA-approved as a food additive, providing stabilizing, thickening, and gelling properties. It has also been used as a sizing agent in the paper and textile industries. Additionally, XG is used in medicinal treatments for diarrhea, dysentery, and colitis and functions as a viscosifier in ophthalmic solutions, as well as in cosmetic and pharmaceutical formulations [4].

Due to its inherently low mechanical strength, XG is often blended with other polymers, such as poly(vinyl alcohol) (PVA), to improve its properties. For instance, XG/PVA hydrogel films have been developed for wound dressing applications, with glycerol added to enhance flexibility and glutaraldehyde (GA) used as a chemical crosslinker. The resulting hydrogel was found to be non-cytotoxic [7,8]. Zhang et al. (2023) demonstrated that a multifunctional electrospun nanofiber dressing composed of XG, PVA, quaternized chitosan, and collagen possessed excellent mechanical strength, antibacterial activity, and biocompatibility. This dressing enhanced cell proliferation and angiogenesis in a mouse model [9]. Despite their abundance, research on XG-based hydrogels remains limited compared to other polysaccharides. However, the inherent biocompatibility of XG offers significant potential for biomedical applications, including wound dressings, drug delivery systems, and tissue engineering.

Hydrogels are three-dimensional polymer networks capable of absorbing and retaining large amounts of water and aqueous solutions. Their network structure allows hydrogels to swell in aqueous environments without dissolving [10]. It should be noted that all hydrogels have molecular-size openings, as defined by the average distance between crosslinks along the polymer chains comprising the hydrogel network (approximately 1–50 nm), also known as mesh size [11]. This mesh size allows for the diffusion of water molecules into hydrogels, as the kinetic diameter of a water molecule is 0.265 nm [10]. In contrast, the hydrogels with interconnected pores beside the available space within the stands of the polymer network are designated as porous or non-dense hydrogels [10,11]. For a dense hydrogel with a closed-cell structure, the swelling rate is very low as it is controlled by the diffusion of water inside the dense polymer network. A non-dense hydrogel can have a significantly higher swelling rate because of capillary action and the decrease in the thickness of polymer domains [10]. Hydrogels with fast swelling rates are advantageous in many applications, such as wound dressings, drug delivery, and environmental remediation. For instance, wound dressings should provide fast absorption of wound exudates. Drug delivery devices should be able to provide rapid drug release under immediate therapeutic needs. For hygienic products such as diapers or hygiene pads, fast-swelling materials provide immediate functionality, thus improving users’ satisfaction. Fast swelling is also beneficial for the improved absorption of contaminated water or removing pollutants.

The incorporation of physical pores into hydrogels has been reported to improve swelling rates significantly, as capillary action leads to extremely fast swelling in open-cell macroporous, hydrophilic material [10,12]. Gas foaming, phase separation, electrospinning, freeze-drying, and templating methods have been used to prepare porous hydrogels [10,11,12]. Freeze-drying or lyophilization produces highly porous structures without the need for organic solvents. Since ice crystals serve as a porogen, this method avoids hydrogel contamination. Pore size can be controlled by adjusting the freezing rate, temperature, polymer concentration, and freezing direction [13]. Freeze-drying is a widely recognized method in biomedical engineering for producing three-dimensional, open-porous medical devices, especially those fabricated from biopolymers [14]. However, achieving optimal mechanical properties and structural integrity remains a significant challenge [10]. Two primary approaches are commonly employed to prepare porous hydrogels via freeze-drying: (1) crosslinking the polymer solution before freeze-drying, or (2) freeze-drying the solution first, followed by crosslinking or co-precipitation. The cooling rate during the freezing step is crucial in determining the final structure. Slow cooling results in a 3D homogeneous structure, whereas rapid cooling, such as quenching in liquid nitrogen or unidirectional freezing, creates a 2D tubular structure with aligned pores [10,15].

This study presents the preparation of fast-swelling hydrogels based on tamarind XG and PVA using citric acid (CA) as a chemical crosslinker. Freeze-drying was used to produce interconnected porous structures. The freeze-dried samples were characterized using Fourier-transform infrared (FTIR) spectroscopy, scanning electron microscopy (SEM), and differential scanning calorimetry (DSC). Porosity was determined using the liquid displacement method. Swelling properties and gel fraction measurements were performed to evaluate the water absorption capacity and crosslinking efficiency of the hydrogels. Cytotoxicity tests were performed to assess the biocompatibility of the hydrogels.

## 2. Materials and Methods

### 2.1. Materials

Tamarind xyloglucan (XG, 95% purity, Mw 800,000 g/mol) was obtained from Megazyme (Bray, Ireland). The polymer composition consisted of xylose, glucose, galactose, arabinose, and other sugar units in a molar ratio of 34:45:17:2:2. Poly(vinyl alcohol) (PVA, Mw 100,000 g/mol, degree of hydrolysis 86–90%) was sourced from Chem-Supply Pty Ltd. (SA, Australia). Citric acid (CA, 99%) was acquired from Ajax Finechem Pty Ltd. (Sydney, NSW, Australia). Glacial acetic acid and sodium hydroxide were purchased from Merck KGaA (Darmstadt, Germany).

### 2.2. Sample Preparation

PVA was dissolved in water and stirred at 80 °C for 20 min using an ultrasonic homogenizer (Vibra-Cell, Sonics & Materials Inc., Newtown, CT, USA). Subsequently, XG was added to the PVA solution and heated at 60 °C for 20 min. CA was then incorporated into the solution. The mixture was poured into a 24-well plate and frozen in a chest freezer (LCDF 220 W, Evermed, Motteggiana, Italy) at −40 °C for 24 h. The frozen samples were then freeze-dried (Alpha 3-4 LSCbasic freeze-dryer, Martin Christ, Osterode am Harz, Germany) at −76 °C and 0.1 mbar pressure for 24 h. The samples obtained were cylindrical in shape. Finally, the samples were crosslinked by placing them on a Teflon tray and heating them in a hot air oven at 120 °C for 30 min. The heating rate was set to 1 °C/min until the temperature reached 145 °C, at which point the crosslinked samples were removed from the oven. The sample designations and formulations are shown in Table 1.

### 2.3. ATR-FTIR Spectroscopy

FTIR spectroscopy (Nicolet iS50, Thermo Fisher Scientific, Waltham, MA, USA) was conducted in the attenuated total reflectance (ATR) mode to identify the functional groups of the freeze-dried samples over a wavenumber range of 4000–450 cm^−1^. The spectra were collected from 64 scans at a resolution of 4 cm^−1^.

### 2.4. Morphological Study

The morphologies of the cross-sections of the prepared samples were analyzed using scanning electron microscopy (JEOL KSM7800F, Akishima, Japan). The freeze-dried samples were fractured in liquid nitrogen and then sputtered with gold prior to analysis.

### 2.5. Differential Scanning Calorimetry (DSC)

Differential scanning calorimetry was conducted using Netzsch DSC 204 F1 Phoenix instrument (Selb, Germany). The samples (5–6 mg) were heated from 25 °C to 375 °C at a rate of 10 °C/min under a nitrogen atmosphere (flow rate: 30 mL/min).

### 2.6. Porosity Determination

The porosity of the samples was assessed following a method adapted from previous reports [16,17]. The procedure involved immersing the samples in EtOH (99.9%, specific gravity) at 25 °C for 30 min, ensuring the samples were fully saturated with EtOH. After immersion, the samples were removed and gently blotted with filter paper to eliminate excess EtOH from their surfaces. They were then immediately weighed using a four-decimal-place balance. The test was conducted in triplicate. The porosity was calculated using the following equation:

(1) $$\mathrm{Porosity}\;(\%)=\frac{W_{2}-W_{1}}{\;\rho_{EtOH}V_{1}}\times 100$$

where *W*_1_ is the weight of the sample before immersing in EtOH, *W*_2_ is the weight of the sample after immersing in EtOH, *V*_1_ is the apparent volume of the sample calculated by measuring their heights and diameters, and ρ*_EtOH_* is the density of EtOH.

### 2.7. Textural Analysis

The hardness of the samples was measured using a texture analyzer (TA-XT2i, Stable Micro Systems, NY, USA). Cylindrical samples, 2 cm in diameter and 2 cm thick, were compressed with a 3.6 cm diameter probe. Compression occurred at a depth of 15 mm at a test speed of 10 mm/s. Hardness was determined by the maximum peak force during the compression cycle, and each measurement was conducted in triplicate.

### 2.8. Determination of Gel Fraction

The samples were accurately weighed to four decimal places and submerged in deionized (DI) water at room temperature for 120 h. Subsequently, they were dried in a hot air oven at 32 °C for 24 h. The gel fraction was determined gravimetrically using the following equation:

(2) $$\mathrm{Gel}\;\mathrm{fraction}=\frac{W_{f}}{W_{i}}\times 100$$

where *W_f_* is the weight of the sample after drying, and *W_i_* is the initial weight before the test.

### 2.9. Swelling Test

The swelling behavior of the dried samples was evaluated at pH 6 using DI water. The samples (80 mg) were submerged in aqueous solution at 27 °C for 48 h. Excess surface water was blotted with filter paper, and the sample was then accurately weighed to four decimal places. The swelling ratio was calculated from three measurements using the following formula:

(3) $$\mathrm{Swelling}\;\mathrm{ratio}\;(\%)=\frac{\left(W_{s}-W_{d}\right)}{W_{d}}\times 100$$

where *W_s_* and *W_d_* represent the weights of the swollen and dried samples, respectively.

### 2.10. Hydrolytic Stability

To evaluate the stability of the hydrogels, PVA and PVA/XG/CA samples were immersed in acetate buffer at pH 5.5, 32 °C (to simulate skin conditions) for nine days. The hydrogels were visually inspected for shape integrity throughout the immersion period.

### 2.11. Cytotoxicity Test

The cytotoxicity of the samples was assessed using mouse fibroblast cells (NCTC 929, 17th passage). The cells were cultured in DMEM supplemented with 10% FBS and 1% antibiotics, seeded into 96-well plates, and incubated at 37 °C in a humidified atmosphere containing 95% air and 5% CO_2_. The hydrogels were UV-sterilized and soaked in a serum-free medium for 24 h. The extraction medium (0.5, 5, and 10 mg/mL) was then applied to the cells for 24 h. Cell viability was determined using the MTT assay, with absorbance measured at 570 nm using a microplate reader. Cells cultured in fresh SFM were used as controls.

## 3. Results and Discussion

### 3.1. FTIR Spectra

**Figure 1:**
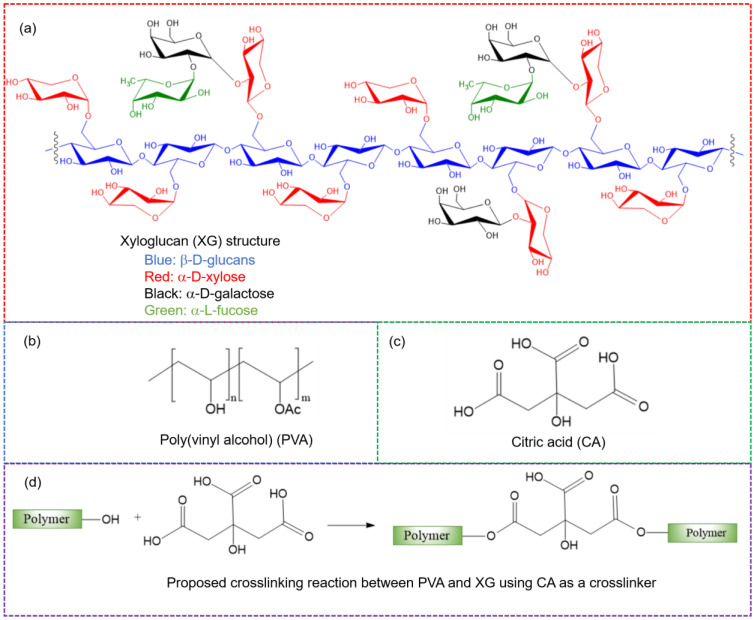
Structural representations of (**a**) tamarind XG [2,5,6], (**b**) partially hydrolyzed PVA [20], (**c**) CA, and (**d**) the crosslinking reaction between PVA and XG with CA as the crosslinker.

**Figure 2:**
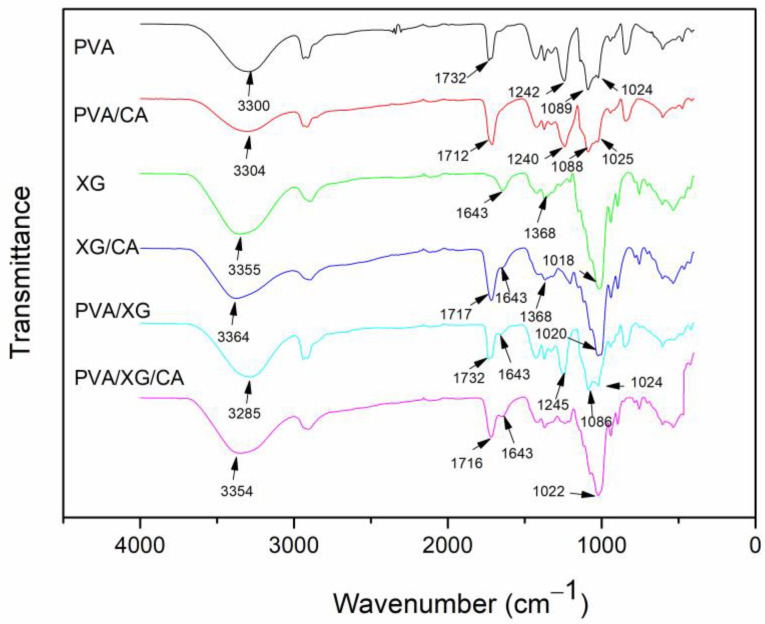
FTIR spectra of the samples before and after crosslinking.

For the interpretation of the FTIR results, the chemical structures of the chemicals used in this study are provided in Figure 1. The FTIR spectra of both the crosslinked and non-crosslinked samples are shown in Figure 2. In the spectrum of PVA, a broad, intense peak at 3300 cm^−1^ is seen, corresponding to O–H stretching and indicative of hydroxyl groups, a feature present in all samples. The peaks at 2939 and 2911 cm^−1^, consistent across all samples, are due to C-H stretching. A notable peak at 1732 cm^−1^ is associated with C=O stretching of the residual acetate groups (–OAc) in partially hydrolyzed PVA due to incomplete hydrolysis [18]. Additionally, the peak at 1089 cm^−1^, characteristic of a secondary alcohol, is attributed to C-O stretching and O-H bending [19].

In the XG spectrum, O-H and C-H stretching peaks are evident at 3355 cm^−1^ and 2895 cm^−1^, respectively. The peak at 1643 cm^−1^ is attributed to absorbed water [21,22], and the strong peak at 1018 cm^−1^ is due to C-O stretching in XG [23]. The spectrum of the PVA/XG blend exhibits all characteristic peaks from each polymer component.

The proposed crosslinking mechanism of PVA and XG with CA, as shown in Figure 1d, indicates noticeable alterations in the FTIR spectra compared with the non-crosslinked samples. The O-H stretching peaks of PVA, XG, and the PVA/XG blends decreased in intensity (relative to other peaks within the same spectrum) and shifted to higher wavenumbers. This decrease in intensity results from the consumption of O–H groups during crosslinking, which also diminishes the extent of hydrogen bonding. As some O–H groups are consumed in the crosslinking process, free O-H groups that are no longer involved in hydrogen bonding are generated, leading to a shift in the O-H peaks to higher wavenumbers. Additionally, the C=O stretching peak, observed initially at 1732 cm^−1^, moves to a lower wavenumber (1712 cm^−1^) after crosslinking with CA, reflecting the formation of new ester groups (–COO–). The newly formed C=O bonds engage in additional hydrogen bonding, prompting a further shift in the C=O absorption peak to lower wavenumbers. This shift is similarly observed in the PVA/XG/CA spectrum.

### 3.2. Morphological Study via Scanning Electron Microscopy (SEM)

The final morphology of the material after freeze-drying is predominantly influenced by the cooling rate. During the cooling process, water transitions through two phases: nucleation in supercooled water and subsequent ice growth. Ice acts as a porogen, and as it forms, polymer chains that are less soluble in ice than in liquid water are excluded, creating a continuous network around the ice crystals. Slow cooling leads to a 3D homogeneous structure, whereas rapid cooling or unidirectional freezing promotes columnar solidification, resulting in a 2D tubular structure with aligned channel pores [15]. Rapid freezing at extremely low temperatures (−196 °C) typically results in smaller, less interconnected pores [13]. Aligned channels have been observed in the material obtained by the directional freezing of 5 wt% aqueous PVA solutions [24]. The temperature gradient causes uneven freezing, where one side solidifies faster than the other, leading to directional ice growth and the formation of aligned channels after ice sublimation.

**Figure 3:**
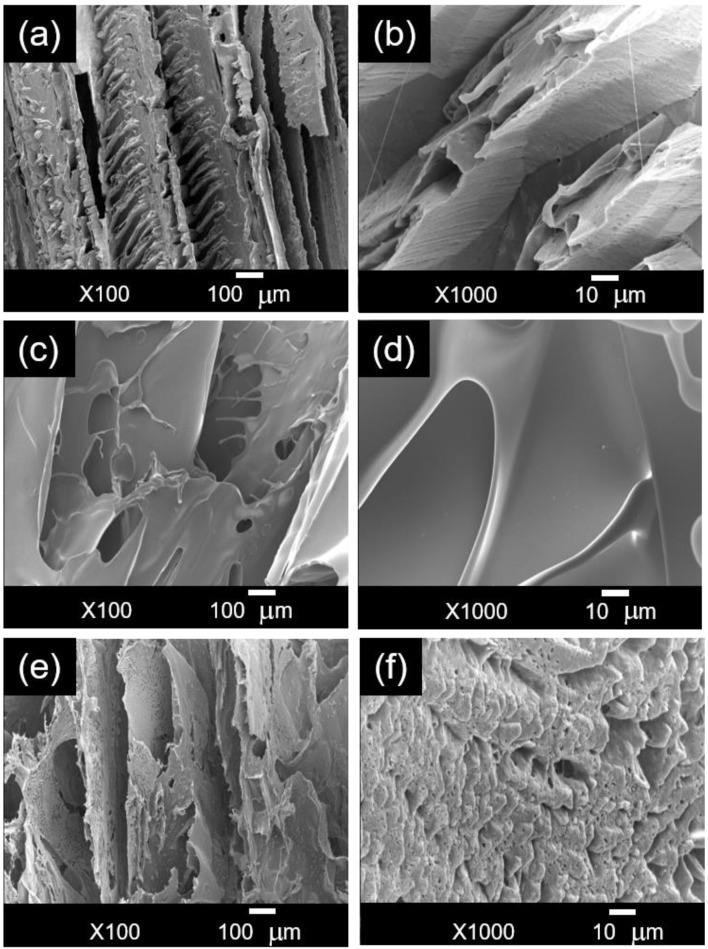
SEM images of the crosslinked samples taken at 100× and 1000× magnifications: (**a**,**b**) PVA/CA, (**c**,**d**) XG/CA, and (**e**,**f**) PVA/XG/CA.

In this study, polymer solutions were dispensed into 24-well polystyrene plates and frozen in a chest freezer at −40 °C for 24 h. This rapid cooling process resulted in the creation of partial longitudinal channels within the crosslinked PVA/CA sample, leading to a hierarchical porous structure and rough surface (Figure 3a,b). The pores varied in size from a few microns to several hundred microns, featuring two distinct sets of channels, larger and smaller, aligned in opposite directions, reflecting the complexity of the ice morphology that developed during the freezing process.

The XG/CA sample shows a distinctly different morphology compared to that of the PVA/CA (Figure 3c,d). The surface of XG/CA appears smooth, resembling a stack of disoriented films with substantial gaps between layers. The pores are irregularly shaped, with sizes ranging from a few hundred micrometers to several millimeters. The crosslinked XG sample exhibits less structured porosity, given that the higher viscosity of the XG solution compared to PVA decreases the mobility of water molecules, delaying nucleation and promoting the formation of larger ice crystals.

Figure 3e,f shows that blending XG with PVA led to a PVA/XG/CA sample with a rougher surface and a less organized porous structure than PVA/CA. This irregularity was probably due to microphase separation during freezing and differences in the thermal properties of the polymers, which led to heterogeneous ice crystal formation. A hierarchical porous structure is also evident in the PVA/XG/CA sample. All samples show interconnected porous structures. Nokoorani et al. reported that chitosan/gelatin-based scaffolds prepared via freeze-drying contained interconnected open pores with a mean pore size in the range of 390–460 μm [16]. The authors claimed that the formation of large pores in their scaffolds was due to the hydrophilicity of both gelatin and chitosan, which led to the formation of large ice crystals in the freeze-drying method.

### 3.3. Porosity Results

Table 2 reveals a significant difference in porosity between the PVA and XG samples. It can be concluded that the porosity in the freeze-dried XG was higher than that of PVA. Blending PVA with 25 wt% XG resulted in a porosity value between the pure polymers. CA crosslinking of PVA or XG samples resulted in a reduction in porosity. This reduction was caused by the formation of covalent bonds between polymer chains during crosslinking, producing a denser polymeric structure. However, crosslinking did not affect the porosity of the PVA/XG sample. This is probably because the phase separation between PVA and XG had the opposite effect on porosity and canceled out the effect of crosslinking. The effects of crosslinking in reducing porosity and pore size have been reported for PVA hydrogels crosslinked with periodate-modified gum Arabic [25]. Nokoorani et al. (2021) prepared freeze-dried chitosan/gelatin-based scaffolds containing allantoin. They found that the porosity values measured from SEM images were in the range of 58.0–63.1%, which were comparable to the values reported in this work. However, the authors claimed that different measurement techniques produced different porosity results; the ethanol displacement technique yielded a porosity range of 21.9–31.9%, which was significantly lower than the results they obtained from SEM [16].

### 3.4. Thermal Properties and Crystallization Behavior

**Figure 4:**
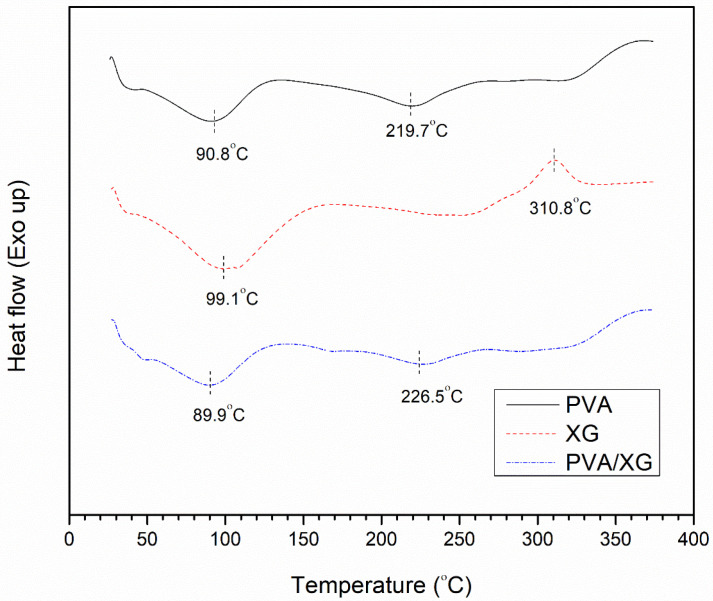
DSC thermograms of the prepared hydrogels.

The DSC plot in Figure 4 shows broad endothermic peaks around 90 °C in all samples, indicative of the evaporation of bound water. This result is expected because PVA and XG are hydrophilic and can form hydrogen bonds with water. An endothermic peak at 219.7 °C is found in the PVA thermogram, corresponding to its melting temperature (T_m_). This finding is consistent with previous studies on crosslinked PVA [25]. It is hypothesized that, during freezing, the partial alignment of the PVA chains leads to the formation of a semi-crystalline structure. The shift in the DSC curve beyond 300 °C signifies the onset of PVA degradation.

In contrast, Figure 4 illustrates that the neat XG sample is amorphous, as no melting peak is present. The decomposition of XG is characterized by an exothermic peak at 310.8 °C. In the PVA/XG blend, the melting peak of the PVA component is less distinct, likely because the presence of XG disrupts the crystallization of PVA.

### 3.5. Swelling Behavior

**Figure 5:**
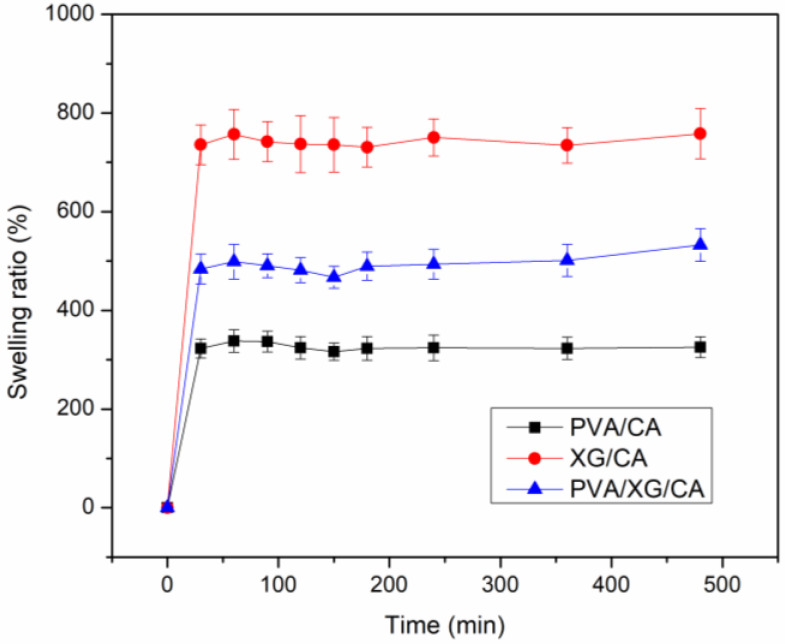
Swelling ratio of the prepared hydrogels.

Figure 5 depicts the fast-swelling behavior of all of the hydrogels, which was completed within 10 s of immersion in DI water. Such fast water absorption was also reported for the hydrogels with aligned macropores, which were obtained using the directional freezing redox polymerization method [26]. Bardajee et al. (2023) developed superporous hydrogels consisting of poly (3-sulfopropyl acrylate-co–N, N-dimethyl ethyl methacrylate-co-acrylamide) grafted onto salep. They observed that the superporous hydrogels required 5.5 min to reach 0.63 of the equilibrium swelling [27].

The swelling ratio values of PVA/CA and XG were 308 ± 19% and 758 ± 51% (Figure 5). The reduced swelling capacity of PVA/CA compared to XG/CA can be attributed to its higher crystallinity and crosslinking density. The PVA/XG/CA hydrogels possessed intermediate swelling behavior, with a swelling ratio of 533 ± 33%. It has been reported that hydrogels with denser inter-polymeric networks had reduced water uptake capacity [28]. Additionally, the addition of XG into chitosan-based hydrogels enhanced their swelling ability due to increased hydrogen bond formation, which promotes greater water absorption [29]. Consistent with these observations, this study found that incorporating XG into the PVA-based hydrogel increased the swelling ratio, whereas the addition of CA decreased it. The swelling ratio values of the hydrogels in this work were comparable with those based on freeze-dried chitosan/pluronic/agarose hydrogels containing interconnected pores of 374–596 μm without the alignment of pores [30].

### 3.6. Gel Fraction

The non-crosslinked samples dissolved completely in water within two hours. However, the crosslinked samples PVA/CA, XG/CA, and PVA/XG/CA were partially dissolved in water. Their gel fractions were 95.88 ± 1.74, 58.32 ± 3.01, and 98.82 ± 0.50, respectively. These findings indicate that CA is more effective for crosslinking PVA than XG. Similarly, previous research has demonstrated that crosslinking XG/PVA with glutaraldehyde increased swelling and yielded very high gel fractions, ranging from 93% to 96% [7].

### 3.7. Hydrolytic Stability Results

After a nine-day incubation of the prepared PVA/XG/CA hydrogels in acetate buffer at pH 5.5 and 32 °C, significant swelling was observed without noticeable disintegration or dissolution.

### 3.8. Textural Analysis Results

**Figure 6:**
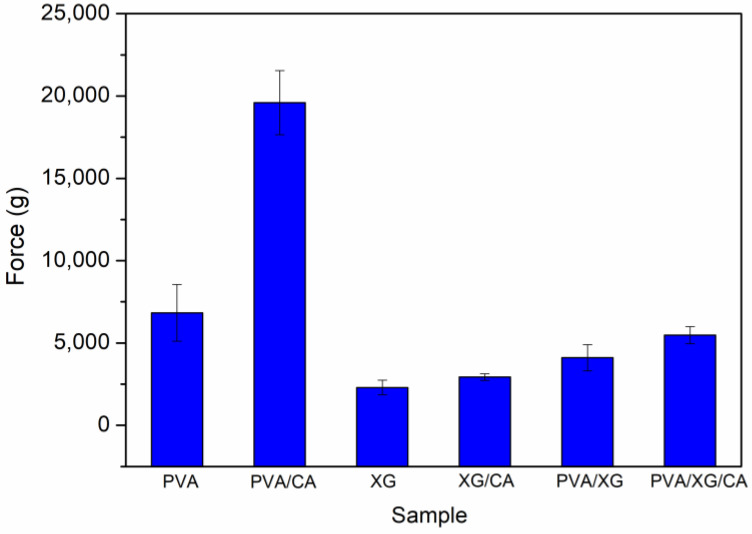
Textural analysis results.

A texture analyzer was used to measure the hardness of the samples, which was defined as the maximum force required to compress them to a predetermined distance at a controlled speed. As illustrated in Figure 6, PVA was found to be more rigid than XG, likely due to its semi-crystalline structure, whereas XG exhibited an amorphous structure, as verified by DSC analysis. After freeze-drying, PVA maintained a more structured configuration than XG. It is well documented that crosslinking enhances the mechanical strength and stiffness of polymers by creating a three-dimensional network, thereby limiting chain mobility [31,32].

Following crosslinking, the hardness of PVA was increased by 187%, which is consistent with its high gel fraction. In contrast, the crosslinking of XG led to only a 28% increase in hardness, reflecting its comparatively lower crosslinking efficiency. Therefore, the hardness of the PVA/XG sample was lower than that of the neat PVA sample. The same trend was observed for their crosslinked counterparts. In summary, although the hardness value of the PVA/XG sample was reduced due to the presence of XG, it was increased by crosslinking.

### 3.9. Cytotoxicity Results

**Figure 7:**
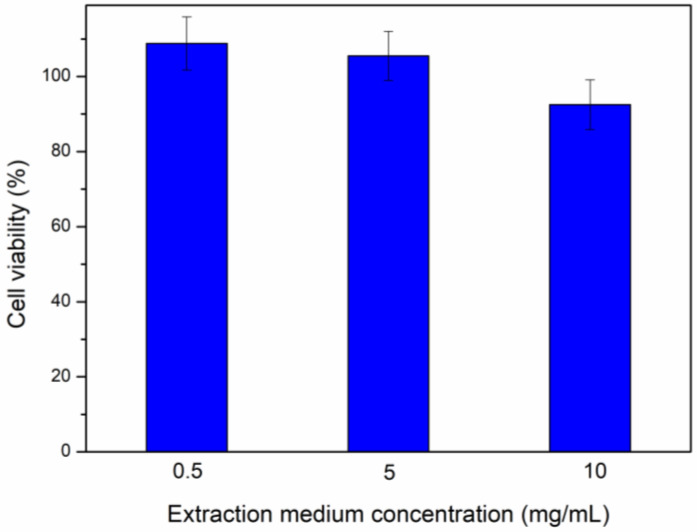
MTT assay results showing cell viability at various extraction medium concentrations.

The PVA/XG/CA sample was selected for cytotoxicity testing because of its comprehensive formulation and superior experimental outcomes. In the cytotoxicity test, NCTC 929 clone cells were cultured with different concentrations of extraction media derived from the hydrogel samples. Figure 7 presents the cell viability results of the MTT assay. According to in vitro cytotoxicity standards, any reduction in cell viability exceeding 30% is considered cytotoxic (ISO 10993 [33]). The viability of the cells exposed to the extraction media was 108.8% ± 7.07, 105.5% ± 6.55, and 92.5% ± 6.59 for extraction medium concentrations of 0.5, 5, and 10 mg/mL, respectively. These results demonstrate that the PVA/XG/CA sample is non-cytotoxic, as the cell viability remained above 70%.

## 4. Conclusions

In this study, tamarind XG and PVA were used to prepare hydrogels and CA was used as a chemical crosslinker. Freeze-drying was employed to generate an open macroporous structure. The pore morphology was anisotropic, implying the partial orientation of PVA molecules during freezing. This orientation was not observed for XG. SEM images of the crosslinked XG/PVA sample revealed irregular pore sizes and surface roughness, suggesting phase separation between PVA and XG. The crosslinking of PVA and XG improved the water stability and hardness of the materials. The gel fraction of PVA was higher than that of XG, indicating that the CA crosslinking of PVA was more efficient than XG. However, the XG sample had a higher porosity value and a higher swelling ratio than those of the PVA sample. In brief, blending XG with PVA provided hydrogels with balanced properties, e.g., a swelling ratio of 533 ± 33% and a porosity of 66.49 ± 1.62%. We observed the rapid swelling of all of the prepared hydrogels due to the extensive interconnected capillary channels, whose sizes were about a few hundred microns. Therefore, all of the prepared hydrogels showed a significantly fast swelling process (within 10 s). Our prepared hydrogels are promising for applications when a fast response is required, e.g., wound dressing and drug delivery. However, the obtained hydrogels still have areas for improvement, including increasing the swelling ratio and enhancing durability for repeated use. A combination of physical and chemical crosslinkers as well as the addition of hydrophilic additives could be used to improve their performance.

## Figures and Tables

**Table 1:** Sample formulation.

Sample	PVA (g)	XG (g)	CA (g)	Water (g)
PVA	4	0	0	96
PVA/CA	4	0	0.8	95.2
XG	0	4	0	96
XG/CA	0	4	0.8	95.2
PVA/XG	3	1	0	96
PVA/XG	3	1	0.8	95.2

**Table 2:** Porosity of the freeze-dried samples.

Sample	Porosity (%)
PVA	63.27 ± 2.08
PVA/CA	56.09 ± 2.79
XG	72.03 ± 2.83
XG/CA	68.99 ± 2.06
PVA/XG	66.36 ± 1.90
PVA/XG/CA	66.49 ± 1.62

## Data Availability

The data presented in this study are available on request from the corresponding author.

## References

[1] Noleto G.R., Petkowicz C.O. (2016). Potential for biomedical applications of galactomannans and xyloglucans from seeds: An overview. Med. Chem..

[2] Gavande P.V., Goyal A., Fontes C.M. (2023). Carbohydrates and carbohydrate-active enZymes (CAZyme): An overview. Glycoside Hydrolases.

[3] Kozioł A., Cybulska J., Pieczywek P.M., Zdunek A. (2015). Evaluation of structure and assembly of xyloglucan from tamarind seed (*Tamarindus indica* L.) with atomic force microscopy. Food Biophys..

[4] Mishra A., Malhotra A.V. (2009). Tamarind xyloglucan: A polysaccharide with versatile application potential. J. Mater. Chem..

[5] Ochoa-Villarreal M., Aispuro-Hernández E., Vargas-Arispuro I., Martínez-Téllez M.Á. (2012). Plant cell wall polymers: Function, structure and biological activity of their derivatives. Polymerization.

[6] Kochumalayil J., Sehaqui H., Zhou Q., Berglund L.A. (2010). Tamarind seed xyloglucan–a thermostable high-performance biopolymer from non-food feedstock. J. Mater. Chem..

[7] Ajovalasit A., Sabatino M.A., Todaro S., Alessi S., Giacomazza D., Picone P., Di Carlo M., Dispenza C. (2018). Xyloglucan-based hydrogel films for wound dressing: Structure-property relationships. Carbohydr. Polym..

[8] Picone P., Sabatino M.A., Ajovalasit A., Giacomazza D., Dispenza C., Di Carlo M. (2019). Biocompatibility, hemocompatibility and antimicrobial properties of xyloglucan-based hydrogel film for wound healing application. Int. J. Biol. Macromol..

[9] Zhang Y.-L., Wang C., Yuan X.-Q., Yan H.-H., Li C.-B., Wang C.-H., Xie X.-R., Hou G.-G. (2023). Multifunctional xyloglucan-containing electrospun nanofibrous dressings for accelerating infected wound healing. Int. J. Biol. Macromol..

[10] Foudazi R., Zowada R., Manas-Zloczower I., Feke D.L. (2023). Porous hydrogels: Present challenges and future opportunities. Langmuir.

[11] De France K.J., Xu F., Hoare T. (2018). Structured macroporous hydrogels: Progress, challenges, and opportunities. Adv. Healthc. Mater..

[12] Zhai N., Wang B. (2023). Preparation of fast-swelling porous superabsorbent hydrogels with high saline water absorbency under pressure by foaming and post surface crosslinking. Sci. Rep..

[13] Qian L., Zhang H. (2011). Controlled freezing and freeze drying: A versatile route for porous and micro-/nano-structured materials. J. Chem. Technol. Biotechnol..

[14] Abdelaziz A.G., Nageh H., Abdo S.M., Abdalla M.S., Amer A.A., Abdal-Hay A., Barhoum A. (2023). A review of 3D polymeric scaffolds for bone tissue engineering: Principles, fabrication techniques, immunomodulatory roles, and challenges. Bioengineering.

[15] Grenier J., Duval H., Barou F., Lv P., David B., Letourneur D. (2019). Mechanisms of pore formation in hydrogel scaffolds textured by freeze-drying. Acta Biomater..

[16] Nokoorani Y.D., Shamloo A., Bahadoran M., Moravvej H. (2021). Fabrication and characterization of scaffolds containing different amounts of allantoin for skin tissue engineering. Sci. Rep..

[17] Deng A., Yang Y., Du S. (2021). Tissue engineering 3D porous scaffolds prepared from electrospun recombinant human collagen (RHC) polypeptides/chitosan nanofibers. Appl. Sci..

[18] Korbag I., Mohamed Saleh S. (2016). Studies on the formation of intermolecular interactions and structural characterization of polyvinyl alcohol/lignin film. Int. J. Environ. Stud..

[19] Pawde S., Deshmukh K. (2008). Characterization of polyvinyl alcohol/gelatin blend hydrogel films for biomedical applications. J. Appl. Polym. Sci..

[20] Gaaz T.S., Sulong A.B., Akhtar M.N., Kadhum A.A.H., Mohamad A.B., Al-Amiery A.A. (2015). Properties and applications of polyvinyl alcohol, halloysite nanotubes and their nanocomposites. Molecules.

[21] Jipa I.M., Stoica A., Stroescu M., Dobre L.-M., Dobre T., Jinga S., Tardei C. (2012). Potassium sorbate release from poly (vinyl alcohol)-bacterial cellulose films. Chem. Pap..

[22] Farias M.D., Albuquerque P.B., Soares P.A., de Sa D.M., Vicente A.A., Carneiro-da-Cunha M.G. (2018). Xyloglucan from *Hymenaea courbaril* var. *courbaril* seeds as encapsulating agent of L-ascorbic acid. Int. J. Biol. Macromol..

[23] Szymanska-Chargot M., Zdunek A. (2013). Use of FT-IR spectra and PCA to the bulk characterization of cell wall residues of fruits and vegetables along a fraction process. Food Biophys..

[24] Zhang H., Hussain I., Brust M., Butler M.F., Rannard S.P., Cooper A.I. (2005). Aligned two-and three-dimensional structures by directional freezing of polymers and nanoparticles. Nat. Mater..

[25] Pandit A.H., Mazumdar N., Imtiyaz K., Rizvi M.M.A., Ahmad S. (2019). Periodate-modified gum arabic crosslinked PVA hydrogels: A promising approach toward photoprotection and sustained delivery of folic acid. ACS Omega.

[26] Zhao D., Zhu J., Zhu Z., Song G., Wang H. (2014). Anisotropic hierarchical porous hydrogels with unique water loss/absorption and mechanical properties. RSC Adv..

[27] Rezanejade Bardajee G., Boraghi S.A., Mahmoodian H., Rezanejad Z., Parhizkari K., Elmizadeh H. (2023). A salep biopolymer-based superporous hydrogel for ranitidine delivery: Synthesis and characterization. J. Polym. Res..

[28] Yacob N., Hashim K. (2014). Morphological effect on swelling behaviour of hydrogel. AIP Conf. Proc..

[29] Martínez-Ibarra D.M., López-Cervantes J., Sánchez-Machado D.I., Sanches-Silva A. (2018). Chitosan and xyloglucan-based hydrogels: An overview of synthetic and functional utility. Chitin-Chitosan—Myriad Functionalities in Science and Technology.

[30] Abdollahi H., Amiri S., Amiri F., Moradi S., Zarrintaj P. (2024). Antibacterial biocomposite based on chitosan/pluronic/agarose noncovalent hydrogel: Controlled drug delivery by alginate/tetracycline beads system. J. Funct. Biomater..

[31] Tanpichai S., Oksman K. (2016). Crosslinked nanocomposite hydrogels based on cellulose nanocrystals and PVA: Mechanical properties and creep recovery. Compos. A Appl. Sci. Manuf..

[32] Bermejo J.S., Ugarte C.M. (2009). Chemical crosslinking of PVA and prediction of material properties by means of fully atomistic MD simulations. Macromol. Theory Simul..

[33] (2018). Biological Evaluation of Medical Devices-Part 1: Evaluation and Testing Within a Risk Management Process.

